# Research Based Elective Placement - A First-hand Student Review

**DOI:** 10.1192/j.eurpsy.2026.11694

**Published:** 2026-07-31

**Authors:** T. Porter, J. Beezhold

**Affiliations:** 1 Norwich Medical School, University of East Anglia, Norwich; 2 Great Yarmouth Acute Services, Northgate Hospital, Great Yarmouth, United Kingdom

## Abstract

**Introduction:**

As per the MMBS course, we are required to partake in an external elective in the fourth year, lasting four weeks. I chose to do my elective under the guidance of a consultant psychiatrist with the goal of participating in research.

**Objectives:**

**Primary Objectives:**

1. One published article as first author
2. One published article as an additional author

**Secondary Objectives**

Assist the consultant in progression of his projects, aiming towards a mutually beneficial elective

1. Enhance my own skillset for my future aspirations as a researcher

**Methods:**

The plan involved writing a poster abstract for a journal, satisfying a first author publication. The other objective would be achieved by working in tandem with the consultant on either finalising a write-up for his concluded project, or by writing up a research protocol.

In addition to this, there were opportunistic experiences during the placement, such as the ability to peer-review two studies awaiting journal publication, and critically appraising two published articles.

**Results:**

2 Journal reviews

2 Critical appraisals

Contributed to finalising a paper for publication

Co-wrote a research protocol

Submitted a poster abstract

**Conclusions:**

The elective offered several experiences and opportunities for me to be involved in a variety of projects, in different aspects of the research process, from forming an idea and writing up a protocol, to reviewing finalised research projects. I believe this experience has enhanced my skillset, offered me unique insight into the world of research and provided a solid foundation to start my research career. This experience has also anecdotally shown me the importance of mentorship in supporting personal growth and development of the skills involved in research.

**Disclosure of Interest:**

None Declared

